# Prolonged Dual Hypothermic Oxygenated Machine Perfusion for Daytime Liver Transplant

**DOI:** 10.1001/jamanetworkopen.2026.5039

**Published:** 2026-04-02

**Authors:** Silke B. Bodewes, Linda C. Woltjes, Adam M. Thorne, Janina Eden, Veerle A. Lantinga, Bianca Lascaris, Hans Blokzijl, Suomi M. G. Fouraschen, Hermien Hartog, Jaap Jan Vos, Ton Lisman, Vincent E. de Meijer

**Affiliations:** 1 Department of Surgery, Section of Hepatobiliary Surgery and Liver Transplantation, University of Groningen, University Medical Center Groningen, Groningen, the Netherlands; 2 UMCG Comprehensive Transplant Center, Groningen, the Netherlands; 3 Department of Gastroenterology and Hepatology, University Medical Center Groningen, University of Groningen, Groningen, the Netherlands; 4 Department of Anesthesiology, University Medical Center Groningen, University of Groningen, Groningen, the Netherlands; 5 Surgical Research Laboratory and Section of Hepatobiliary Surgery and Liver Transplantation, Department of Surgery, University of Groningen, University Medical Center Groningen, Groningen, the Netherlands

## Abstract

**Importance:**

Liver transplants are performed around the clock, often associated with substantial disutility for patients and clinicians. While short-duration dual hypothermic oxygenated machine perfusion (short-DHOPE) mitigates ischemia-reperfusion injury and related complications, prolonged DHOPE (DHOPE-PRO) may further extend preservation time and facilitate daytime liver transplant.

**Objective:**

To assess whether the use of DHOPE-PRO is associated with an increased proportion of daytime liver transplants without compromising graft or patient outcomes.

**Design, Setting, and Participants:**

This prospective cohort study conducted at a large academic liver transplant center in the Netherlands included adult and pediatric recipients of liver grafts received from donation after brain death (DBD), donation after circulatory death (DCD), or living donors. The study compared all liver transplants performed between January 1, 2023, and December 31, 2024, following routine DHOPE-PRO implementation, with a control cohort of all consecutive liver transplants performed between January 1, 2021, and December 31, 2022. Follow-up continued through December 31, 2025. Outcomes were stratified by graft type.

**Exposures:**

Grafts underwent DHOPE-PRO, with or without viability assessment depending on graft risk, or short-DHOPE (for DCD livers) or no perfusion (for DBD livers).

**Main Outcomes and Measures:**

The primary outcome was the difference in the percentage of daytime liver transplants, defined as surgery starting at or after 8 am and either (1) reperfusion occurring before 8 pm or (2) completion by midnight, between the 2021-2022 and 2023-2024 cohorts. Secondary outcomes included 1-year patient and graft survival, intraoperative parameters, and postoperative complications, stratified by graft type.

**Results:**

A total of 330 liver transplants (median [IQR] age, 45 [13-62] years; 186 [56.4%] male) were included in the analyses: 155 transplants in the 2021-2022 cohort and 175 transplants in the 2023-2024 cohort. Following DHOPE-PRO implementation, the percentage of daytime transplants increased from 48.4% (75 of 155) to 84.6% (148 of 175) for reperfusion (*P* < .001) and from 53.5% (83 of 155) to 89.1% (156 of 175) for completion (*P* < .001). Median (IQR) DHOPE duration increased from 2.1 (1.6-4.1) to 10.2 (5.1-13.1) hours (*P* < .001), with total preservation times up to 31.4 hours. Use of DHOPE-PRO showed no association with postoperative complications (eg, new-onset acute kidney injury; odds ratio, 0.64 [95% CI, 0.37-1.07]; *P* = .09]). One-year patient survival exceeded 90% in all subgroups. Use of DHOPE-PRO was not associated with either graft (hazard ratio, 1.28 [95% CI, 0.59-2.74]; *P* = .53) or patient (hazard ratio, 2.05 [95% CI, 0.75-5.59]; *P* = .16) survival.

**Conclusions and Relevance:**

In this prospective cohort study, routine implementation of DHOPE-PRO was associated with increased proportions of daytime liver transplants and with improved surgical logistics. It was also associated with outcomes similar to those after short-duration DHOPE, supporting its broader application in clinical practice.

## Key Points

**Question:**

Is routine application of prolonged dual hypothermic oxygenated machine perfusion (DHOPE-PRO) associated with an increased proportion of daytime liver transplants?

**Findings:**

In this cohort study of 330 liver transplants, the percentage of daytime liver transplants increased from 53% to 89%, with graft preservation times extended up to 31 hours, after routine implementation of DHOPE-PRO.

**Meaning:**

The findings suggest that use of DHOPE-PRO is associated with an increased proportion of daytime liver transplants and with improved surgical logistics.

## Teaser

This cohort study investigates whether the use of prolonged dual hypothermic oxygenated machine perfusion is associated with increased daytime liver transplants among adult and pediatric recipients.

## Introduction

Machine perfusion has advanced the field of liver transplantation by improving organ preservation and enabling preimplementation graft viability assessment. Currently, 2 ex situ machine perfusion strategies predominate clinical practice: dual hypothermic oxygenated machine perfusion (DHOPE) and normothermic machine perfusion (NMP).^1,2^

DHOPE preserves the donor liver at around 10 °C with active oxygenation via both the portal vein and hepatic artery. A short period (1-2 hours) of DHOPE prior to transplant mitigates ischemia-reperfusion injury by preserving mitochondrial function and maintaining hepatic nucleotide stores.^3^ Randomized clinical trials have demonstrated that DHOPE reduces the incidence of biliary complications, early allograft dysfunction, postreperfusion syndrome, and other transplant-related complications,^4,5,6,7,8,9,10^ and data outside randomized clinical trial settings have shown excellent long-term outcomes.^11^

NMP maintains the donor liver at 35 to 37 °C, with correspondingly higher vascular pressures and flow rates. This method supports ongoing metabolic activity and enables real-time functional assessment of the graft. Therefore, NMP is particularly beneficial for adequate selection of grafts from extended criteria donors or grafts exposed to substantial ischemic injury. Improved selection of these grafts has been shown to expand the pool of transplantable donor livers.^12,13,14,15,16^

In clinical practice, the choice between perfusion strategies largely depends on donor type and graft quality.^17^ In the Netherlands, livers from donation after circulatory death (DCD) donors routinely undergo end-ischemic short-duration DHOPE, with selective addition of NMP for viability assessment in high-risk, so called extended criteria donor (ECD), grafts.^18^ Donation after brain death (DBD) livers have traditionally been preserved using static cold storage.

**Figure 1.**
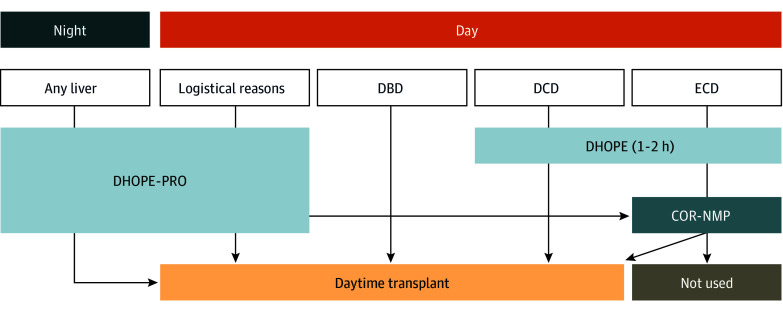
Schematic Overview of the Allocation of Machine Perfusion Strategies (2023-2024) Based on Time of Arrival Following Implementation of Routine Prolonged Dual Hypothermic Oxygenated Machine Perfusion (DHOPE-PRO) to Facilitate Daytime Liver Transplants During nighttime hours, all livers were routinely placed on DHOPE-PRO to facilitate daytime transplant. Donation after brain death (DBD) grafts arriving during the day may be transplanted without machine perfusion depending on logistical and clinical factors. Standard-risk donation after circulatory death (DCD) grafts underwent DHOPE or DHOPE-PRO based on time of arrival. Extended criteria donor ECD grafts underwent DHOPE or DHOPE-PRO based on the time of arrival followed by controlled oxygenated rewarming and normothermic machine perfusion (COR-NMP) for graft viability assessment.

Given that short-duration DHOPE is associated with reduced posttransplant biliary complications, lower costs, and the ability to preserve donor livers at substantially lower perfusion pressures and flow rates compared with NMP, members of our team investigated in preclinical and observational studies the possibility of DHOPE to prolong preservation time, referred to as DHOPE-PRO.^19,20^ Building on the findings in these studies, members of our team conducted the pioneering clinical DHOPE-PRO trial, demonstrating that DHOPE-PRO is both safe and feasible to enable daytime liver transplant.^21^ As a result, routine application of DHOPE-PRO was implemented at the University Medical Center Groningen (UMCG) Comprehensive Transplant Center in January 2023 to avoid nighttime transplants (Figure 1).

Because the original DHOPE-PRO trial focused on DBD livers and assessed feasibility and short-term safety, the broader clinical impact of routine DHOPE-PRO use across all donor types and recipient groups remains to be established. Given the potential implications for surgical logistics and transplant outcomes, a comprehensive evaluation with longer follow-up is necessary.

In the current prospective cohort study, we analyzed the implementation of DHOPE-PRO compared with a historical observational cohort from the 2 years immediately preceding implementation. We hypothesized that routine use of DHOPE-PRO would be associated with a significantly higher proportion of daytime liver transplants, without compromising graft or patient outcomes.

## Methods

### Study Design and Population

This cohort study is an interim analysis of a single-center, prospective, observational cohort study and a nonrandomized clinical trial (DHOPE-PRO-LONG) listed with ClinicalTrials.gov.^22^ All consecutive adult and pediatric liver transplants performed between January 1, 2023, and December 31, 2024, were included. No exclusion criteria were applied. Outcomes were compared with a historical cohort of consecutive transplants conducted between January 1, 2021, and December 31, 2022. Data were prospectively collected through the UMCG TransplantLines Biobank and cohort study,^23^ which was approved by the institutional review board of UMCG. Written informed consent was obtained from all patients prior to inclusion. The study was conducted in accordance with the Declarations of Helsinki^24^ and Istanbul, and results were reported according to the Strengthening the Reporting of Observational Studies in Epidemiology (STROBE) reporting guideline.^25^

### Outcomes

The primary outcome was the difference in the proportion of daytime liver transplants between the 2021-2022 and 2023-2024 cohorts, defined as surgery starting at or after 8 am and either (1) reperfusion occurring before 8 pm or (2) completion of the surgical procedure by midnight. Secondary outcomes included graft survival, patient survival, intraoperative parameters, and postoperative complications, stratified by graft type. Three graft type subgroups were analyzed by their conventional preservation strategies before DHOPE-PRO implementation to assess the clinical associations of DHOPE-PRO across different graft types. For DBD grafts, outcomes were compared between grafts preserved with static cold storage (no perfusion) and those treated with DHOPE-PRO. For standard DCD grafts, we compared outcomes between short-duration DHOPE and DHOPE-PRO. ECD-DCD grafts (ie, donor age >60 years, functional warm ischemia time >30 minutes, or donation after euthanasia) were nationwide declined for up-front transplant and were mandated viability assessment by national protocol. For ECD-DCD grafts, outcomes were compared between short-duration DHOPE followed by controlled oxygenated rewarming (COR) and NMP (DHOPE-COR-NMP) and DHOPE-PRO followed by COR-NMP (DHOPE-PRO-COR-NMP).

### Definitions

Posttransplant complications included postreperfusion syndrome (≥30% decrease in mean arterial pressure lasting ≥1 minute within 5 minutes after reperfusion),^26^ primary nonfunction (graft failure without identifiable technical or immunologic cause leading to retransplant or death within 7 days), early allograft dysfunction (total bilirubin levels ≥10 mg/dL [to convert to µmol/L, multiply by 17.104] or international normalized ratio ≥1.6 on postoperative day 7, or alanine aminotransferase [ALT] or aspartate aminotransferase [AST] levels >2000 U/L [to convert AST or ALT to µkat/L, multiply by 0.0167] within the first 7 days),^27^ vascular complications (bleeding, hepatic artery thrombosis, or portal vein thrombosis), biliary complications (nonanastomotic strictures, anastomotic strictures, and bile leakage), acute kidney injury (according to KDIGO [Kidney Disease: Improving Global Outcomes] criteria within 7 days),^28^ and the need for new-onset renal replacement therapy (RRT). All complications within the first year were graded according to the Clavien-Dindo classification.^29^

### Machine Perfusion Procedures

Perfusions were performed using the Liver Assist device (XVIVO), following the protocol previously published by members of our team.^18^ In grafts selected for viability assessment, DHOPE was followed by 1 hour of COR and at least 2.5 hours of NMP. Hepatobiliary viability was evaluated after 2.5 hours of NMP using predefined criteria, as previously described.^18^ Livers accepted for transplant continued receiving NMP until implantation.

### Statistical Analysis

Descriptive statistics were used to summarize donor, perfusion, and recipient demographics as well as clinical and transplant-related variables. For comparisons between groups, categorical variables were compared using the χ^2^ test. Continuous variables were compared using the 2-sample unpaired *t* test. Variables were summarized by group as counts with percentages for categorical data and as medians with IQRs for continuous data. A 2-sided *P* < .05 was considered statistically significant. Graft and patient survival data are presented as actuarial data estimated by Kaplan-Meier survival analysis. Time zero was the date of transplant, and follow-up continued until retransplant or death. Participants without an event were censored at the last recorded follow-up or at administrative censoring on December 31, 2025, whichever occurred first, ensuring a minimum follow-up of 1 year for each patient. Missing data were infrequent (<5%) across study variables and were not imputed. To examine factors associated with new-onset acute kidney injury (AKI) after transplant and the need for new-onset RRT, including the role of prolonged machine perfusion, we first screened variables using univariable logistic regression, and then entered the significant variables into multivariable models. Following the same approach as for new-onset AKI and RRT, we fitted multivariable Cox proportional hazards regression for graft and patient survival. All analyses were performed in R, version 4.3.1 (R Project for Statistical Computing).

## Results

### Study Population

Between January 1, 2021, and December 31, 2024, a total of 372 livers were included, comprising 127 from DBD donors, 171 from DCD donors, and 74 from living donors. Of these, 42 livers (1 DBD, 41 DCD) were not transplanted following viability assessment using the DHOPE-COR-NMP protocol, resulting in a total of 330 transplanted grafts (median [IQR] age, 45 [13-62 years]; 144 [43.6%] female, 186 [56.4%] male) (eFigure 1 in Supplement 1). Liver use rates after viability assessment were 69.6% in the 2021-2022 cohort and 65.6% in the 2023-2024 cohort (*P* = .67). Donor and perfusion characteristics were comparable between the 2021-2022 cohort (n = 155) and the 2023-2024 cohort (n = 175, of which 1 was a split graft) (eTable in Supplement 1).

### Primary Outcome: Daytime Liver Transplant

Following routine implementation of DHOPE-PRO in 2023, the percentage of patients receiving a donor liver preserved with DHOPE-PRO significantly increased from 14.2% (22 of 155) in the 2021-2022 cohort to 51.7% (90 of 174) in the 2023-2024 cohort (*P* < .001). The median (IQR) duration of DHOPE significantly increased from 2.1 (1.6-4.1) hours in the 2021-2022 cohort to 10.2 (5.1-13.1) hours in the 2023-2024 cohort (*P* < .001). The duration of the COR-NMP phase remained consistent across both periods, with median [IQR] values of 9.4 (8.3-10.7) and 9.2 (8.6-9.9) hours, respectively (*P* = .27). Consequently, the median (IQR) total machine perfusion time increased from 7.8 (2.6-10.7) to 14.3 (7.8-19.3) hours (*P* < .001), and median (IQR) total preservation time increased from 9.3 (7.3-15.3) to 18.6 (9.1-24.6) hours (*P* < .001). The maximum DHOPE duration was 20.5 hours for DBD, 23.8 hours for standard DCD, and 13.9 hours followed by COR-NMP for ECD-DCD. The corresponding maximum total preservation times were 26.3 hours for DBD, 27.8 hours for standard DCD, and 31.4 hours for ECD-DCD livers.

**Figure 2.**
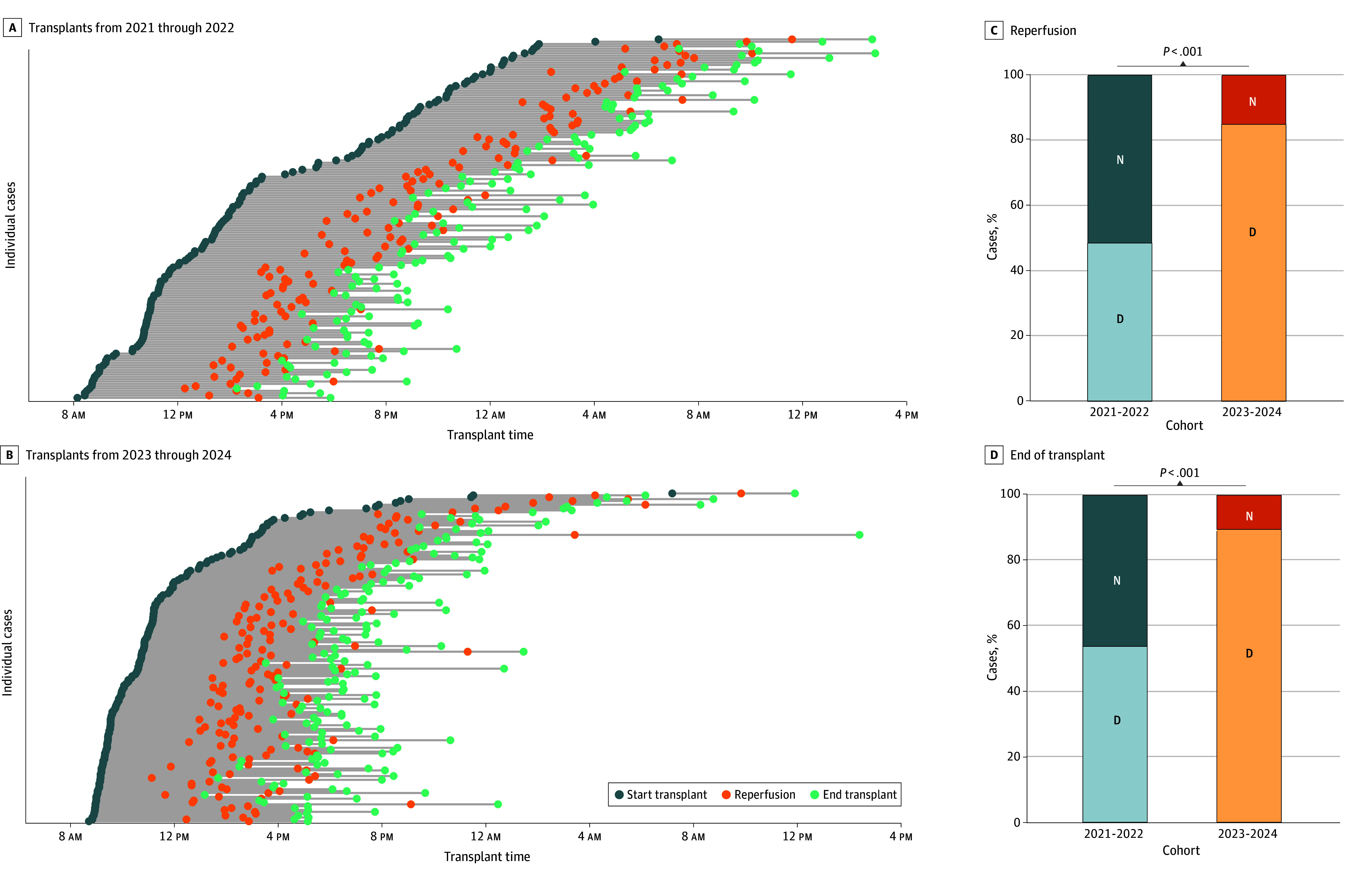
Range Plots and Bar Graphs of Distribution of Transplant Times Before (2021-2022) and After (2023-2024) Implementation of Routine Prolonged Dual Hypothermic Oxygenated Machine Perfusion For reperfusion, D indicates daytime (8 am to 8 pm); N, nighttime (8 pm to 8 am). For end of transplant, D indicates daytime (8 am to midnight); N, nighttime (midnight to 8 am).

In parallel, compared with 2021 through 2022 (Figure 2A), a significant shift in the timing of liver transplant procedures was observed toward daytime hours in 2023 through 2024 (Figure 2B). In 2021 through 2022, graft reperfusion occurred during daytime hours in 48.4% of cases (75 of 155) and during nighttime hours in 51.6% (80 of 155) of cases (Figure 2C), while liver transplant procedures concluded during the day in 53.5% of cases (83 of 155) and at night, in 46.5% of cases (72 of 155) (Figure 2D). In 2023 through 2024, the percentage of daytime reperfusions significantly increased to 84.6% (148 of 175) (*P* < .001) (Figure 2C), and the percentage of procedures completed during daytime hours increased to 89.1% (156 of 175) (*P* < .001) (Figure 2D). Consistent with the implementation strategy to permit daytime transplant, perfusion activity more often occurred overnight; when applicable, COR-NMP was preferentially initiated between 3 am and 4 am to enable early-morning viability assessment (eFigure 2 in Supplement 1).

**Figure 3.**
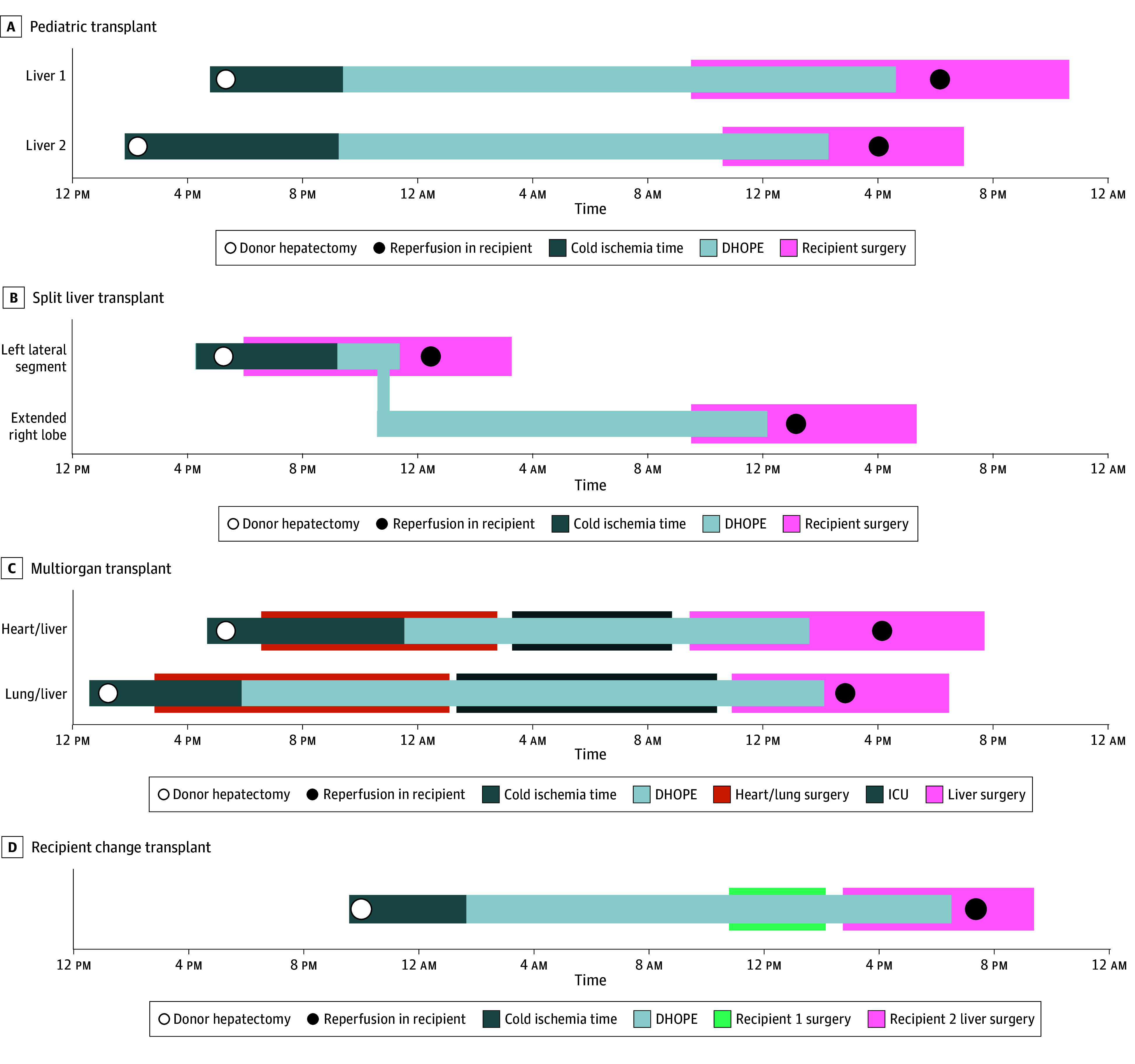
Timelines Depicting Clinical Applications of Prolonged Dual Hypothermic Oxygenated Machine Perfusion (DHOPE-PRO) in Complex Transplant Scenarios A, Timelines correspond to 2 pediatric cases, illustrating the transplant processes and the role of DHOPE-PRO in extending preservation. B, Liver graft that underwent an ex situ split procedure during DHOPE. The left lateral segment was transplanted immediately after splitting, whereas the extended right lobe continued receiving DHOPE-PRO until the next morning. C, Grafts continuing to receive DHOPE-PRO during combined heart-liver or lung-liver transplant procedures to avoid additional ischemic injury. After stabilization and weaning from extracorporeal support in the intensive care unit (ICU), liver transplant was performed the following morning. D, Graft initially accepted for a patient with hilar cholangiocarcinoma. However, after exploration, progressive disease was confirmed by biopsy. The liver was reallocated to the designated backup recipient and remained viable due to continuous DHOPE-PRO.

In 2023 through 2024, the use of DHOPE-PRO was associated with increased logistical flexibility toward the surgical options and timing of complex transplant cases, such as late retransplant or complex portal vein thrombosis in pediatric recipients (Figure 3A and eFigure 3 in Supplement 1), sequential (pediatric) split-liver transplant (Figure 3B), combined heart-liver or lung-liver transplant (Figure 3C), and reallocation of a donor liver due to necessary but unforeseen change in recipient (Figure 3D).

### Secondary Outcomes by Graft Type

The Table details recipient characteristics and postoperative outcomes across 3 subgroups: (1) DBD grafts (no machine perfusion vs DHOPE-PRO); (2) standard DCD grafts (DHOPE vs DHOPE-PRO); and (3) ECD-DCD grafts requiring viability assessment (DHOPE-COR-NMP vs DHOPE-PRO-COR-NMP). Recipient characteristics were largely comparable within each subgroup. However, in the DBD subgroup, recipients of nonperfused grafts had a significantly higher median (IQR) laboratory model for end-stage liver disease (MELD) score compared with recipients of DHOPE-PRO grafts (23 [12-30] vs 14 [10-21]; *P* = .01).

**Table.** Recipient Characteristics and Posttransplant Outcomes Stratified by Graft Type

Characteristic or outcome	DBD		*P* value	Standard DCD		*P* value	ECD-DCD (viability assessed)		*P* value
	No machine perfusion (n = 52)	DHOPE-PRO (n = 52)		DHOPE (n = 34)	DHOPE-PRO (n = 22)		DHOPE- COR-NMP (n = 35)	DHOPE-PRO- COR-NMP (n = 36)	
Age, median (IQR), y	43 (21-56)	47 (23-60)	.64	55 (34-65)	59 (47-67)	.09	61 (47-66)	58 (52-65)	.99
Sex, No. %									
Female	22 (42.3)	18 (34.6)	.55	13 (38.2)	11 (50.0)	.55	14 (40.0)	15 (41.7)	>.99
Male	30 (57.7)	34 (65.4)		21 (61.8)	11 (50.0)		21 (60.0)	21 (58.3)	
BMI, median (IQR)	24 (22-28)	23 (20-29)	.58	25 (22-28)	27 (24-29)	.19	27 (25-31)	27 (24-30)	.79
Laboratory MELD score, median (IQR)	23 (12-30)	14 (10-21)	.01	16 (8-21)	13 (11-20)	.89	12 (8-18)	14 (8-16)	.71
Transplant indication, No. %									
Cirrhosis			.58			.61			.52
MASH	4 (7.7)	2 (3.8)		1 (2.9)	0		0	2 (5.6)	
Other	3 (5.8)	2 (3.8)		4 (11.8)	2 (9.1)		5 (14.3)	2 (5.6)	
Postalcoholic	0	1 (1.9)		3 (8.8)	1 (4.5)		4 (11.4)	4 (11.1)	
Viral	0	1 (1.9)		1 (2.9)	0		2 (5.7)	2 (5.6)	
Acute hepatic failure	12 (23.1)	6 (11.5)		1 (2.9)	1 (4.5)		0	0	
Cancer	7 (13.5)	10 (19.2)		7 (20.6)	6 (27.3)		8 (22.9)	9 (25.0)	
Cholestatic disease	19 (36.5)	19 (36.5)		12 (34.3)	7 (31.2)		9 (25.7)	5 (13.9)	
Congenital biliary disease	1 (1.9)	3 (5.8)		3 (8.8)	0		0	0	
Congenital metabolic disease	2 (3.8)	1 (1.9)		1 (2.9)	1 (4.5)		2 (5.7)	2 (5.6)	
Polycystic disease	1 (1.9)	4 (7.7)		1 (2.9)	2 (9.1)		5 (14.3)	8 (22.2)	
Other	3 (5.8)	3 (5.8)		0	2 (9.1)		0	2 (5.6)	
Operation duration, median (IQR), h	8.3 (6.9-9.2)	8.2 (7.1-9.8)	.70	8.2 (7.6-9.4)	8.4 (7.8-9.1)	.49	8.3 (7.6-9.0)	8.2 (7.6-9.0)	.59
Blood loss, median (IQR), mL	2000 (900-3388)	1650 (850-3750)	.70	2500 (1700-3900)	2125 (550-3450)	.60	2500 (1500-4350)	1700 (1000-3175)	.97
RBCs, median (IQR), units	3 (1-5)	3 (0-6)	.60	3 (1-6)	2 (0-5)	.43	3 (1-6)	2 (0-3)	.67
Postreperfusion syndrome, No. (%)	2 (3.8)	6 (11.5)	.27	3 (8.8)	2 (9.1)	>.99	4 (11.4)	4 (11.1)	>.99
ICU stay, median (IQR), h	40 (24-109)	42.5 (22-92)	.88	36 (18-42)	44 (39-98)	.22	31 (23-43)	30 (21-44)	.45
Hospital stay, median (IQR), d	14 (11-20)	14 (10-26)	.23	16 (11-21)	21 (11-22)	.22	13 (9-18)	14 (101-17)	.35
Primary nonfunction, No. (%)	0	0	>.99	2 (5.9)	1 (4.5)	>.99	0	0	>.99
Early allograft dysfunction, No. (%)	14 (26.9)	12 (23.1)	.66	13 (38.2)	8 (36.4)	>.99	3 (8.6)	9 (25.0)	.14
Hepatic artery thrombosis, No. (%)	0	2 (3.8)	.48	2 (5.9)	1 (4.5)	>.99	3 (8.6)	2 (5.6)	.97
Portal vein thrombosis, No. (%)	0	1 (1.9)	>.99	1 (2.9)	0	>.99	0	1 (2.8)	>.99
Anastomotic biliary stricture, No. (%)									
6 mo	11 (21.1)	12 (23.1)	.56	12 (35.3)	6 (27.7)	.25	11 (31.4)	12 (33.3)	.36
12 mo	12 (23.1)	15 (28.8)	.66	15 (44.1)	6 (27.7)	.32	13 (37.1)	13 (36.1)	.43
Nonanastomotic biliary stricture, No. (%)									
6 mo	0	0	>.99	6 (17.6)	3 (13.6)	.21	3 (8.6)	2 (5.6)	.97
12 mo	0	1 (1.9)	>.99	8 (23.5)	3 (13.6)	.30	3 (8.6)	2 (5.6)	.97
New-onset AKI, No. (%)	14 (26.9)	21 (40.4)	.21	15 (44.1)	13 (59.1)	.41	8 (22.9)	12 (33.3)	.47
Stage 1	4 (7.7)	6 (11.5)	.34	3 (8.8)	4 (18.2)	.69	3 (8.6)	4 (11.1)	.92
Stage 2	7 (13.5)	6 (11.5)		4 (11.8)	2 (9.1)		3 (8.6)	4 (11.1)	
Stage 3	3 (5.8)	9 (17.3)		8 (23.5)	7 (31.8)		2 (5.7)	4 (11.1)	
AKI recovery, median (IQR), d	7 (7-13)	4 (3-8)	.49	6 (3-16)	7 (4-23)	.49	4 (4-5)	4 (3-9)	.56
AKI severity, No. (%)									
1-2 d	1 (1.9)	3 (5.8)	.27	2 (5.9)	1 (4.5)	.86	1 (2.9)	1 (2.8)	.56
3-7 d	6 (11.5)	12 (23.1)		6 (17.6)	5 (22.7)		6 (17.1)	7 (19.4)	
>7 d	6 (11.5)	6 (11.5)		7 (20.6)	6 (27.3)		1 (2.9)	4 (11.1)	
New-onset RRT, No. (%)									
Immediately postoperative	2 (3.8)	5 (9.6)	.29	1 (2.9)	5 (22.7)	.06	1 (2.9)	1 (2.8)	>.99
3 mo	0	1 (1.9)	.23	0	0	>.99	0	0	>.99
6 mo	0	1 (1.9)	.23	0	0	>.99	0	0	>.99
Reoperation, cause, No. (%)	7 (13.5)	12 (23.1)		11 (32.4)	8 (36.4)		10 (28.6)	10 (27.8)	
Bleeding	5 (9.6)	5 (9.6)	.31	4 (11.8)	4 (18.2)	.98	3 (8.6)	3 (8.3)	>.99
Biliary	1 (1.9)	3 (5.8)		0	1 (4.5)		3 (8.6)	2 (5.6)	
Vascular	0	4 (7.7)		3 (8.8)	2 (9.1)		2 (5.7)	0	
Other	1 (1.9)	0		4 (11.8)	1 (4.5)		2 (5.7)	5 (13.9)	
Complications, Clavien-Dindo ≥IIIb, No. (%)	11 (21.2)	16 (30.8)	.26	12 (35.3)	11 (50.0)	.25	8 (22.9)	10 (27.8)	.43
Graft survival, No. (%)									
6 mo	47 (90.4)	49 (94.2)	.71	30 (88.2)	20 (90.9)	.70	34 (97.1)	32 (88.9)	.37
12 mo	47 (90.4)	49 (94.2)	.71	28 (82.4)	19 (86.4)	.98	33 (94.3)	32 (88.9)	.70
Patient survival, No. (%)									
6 mo	47 (90.4)	50 (96.2)	.43	33 (97.1)	20 (90.9)	.70	35 (100)	33 (91.7)	.25
12 mo	47 (90.4)	50 (96.2)	.43	33 (97.1)	20 (90.9)	.70	35 (100)	33 (91.7)	.25
Retransplant, No. (%)	1 (1.9)	1 (1.9)	>.99	6 (17.6)	1 (4.5)	.30	2 (5.7)	1 (2.8)	.98

Abbreviations: AKI, acute kidney injury; ALT, alanine aminotransferase; AST, aspartate aminotransferase; BMI, body mass index (calculated as weight in kilograms divided by height in meters squared); COR, controlled oxygenated rewarming; DBD, donation after brain death; DCD, donation after circulatory death; DHOPE, dual hypothermic oxygenated machine perfusion; DHOPE-PRO, prolonged DHOPE; ECD, extended criteria donor; ICU, intensive care unit; MASH, metabolic dysfunction-associated steatohepatitis; MELD, model for end-stage liver disease; NMP, normothermic machine perfusion; RBCs, red blood cells; RRT, renal replacement therapy.

**Figure 4.**
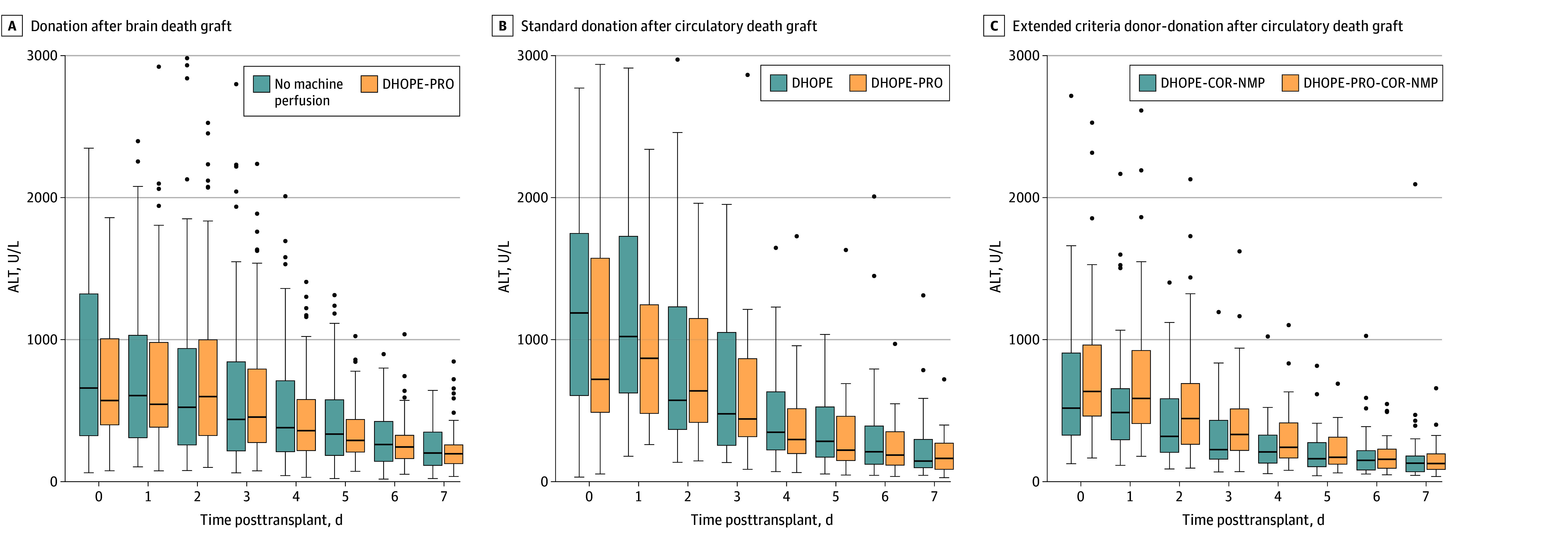
Box and Whisker Plots of Alanine Transferase (ALT) Levels After Transplant Horizonal line in the box indicates median; box edges, IQRs; error bars, minimum and maximum excluding outliers; data points, outliers. COR represents controlled oxygenated rewarming; DHOPE, dual hypothermic oxygenated machine perfusion; DHOPE-PRO, prolonged DHOPE; and NMP, normothermic machine perfusion. To convert ALT to microkatals, multiply by 0.0167.

No significant differences were observed in intraoperative parameters, including duration of the operation, blood loss, transfusion requirements, or incidence of postreperfusion syndrome. Postoperative intensive care unit stay and hospital length of stay were also comparable between groups (Table). Early postoperative ALT levels (Figure 4) and AST levels (eFigure 4 in Supplement 1) were largely similar between groups. Peak ALT and AST levels did not correlate with DHOPE preservation duration in any subgroup (eFigure 4 in Supplement 1).

Outcomes after transplant were comparable across groups. In the standard DCD subgroup, more patients required immediate postoperative RRT in the DHOPE-PRO group compared with the short-duration DHOPE group (5 patients [22.7%] vs 1 patient [2.9%]; *P* = .06). AKI severity (ie, duration of 1-2 days, 3-7 days, or >7 days) and median time until AKI recovery, however, were comparable between groups (Table). Laboratory MELD score, blood loss, and operation duration were each significant in univariable analyses for new-onset AKI and new-onset RRT. In the multivariable models, only the laboratory MELD score remained independently associated with new-onset AKI (odds ratio [OR], 0.96 [95% CI, 0.93-0.99]; *P* = .01) and with new-onset RRT (OR, 0.94 [95% CI, 0.90-0.98]; *P* < .001). Use of DHOPE-PRO was not associated with new-onset AKI (OR, 0.64 [95% CI, 0.37-1.07]; *P* = .09) or new-onset RRT (OR, 0.60 [95% CI, 0.26-1.34]; *P* = .21) (eFigure 5 in Supplement 1).

Graft and patient survival at 6 and 12 months were similar across all subgroups, with overall 1-year patient survival rates at or above 90% (Table). For univariable analysis, laboratory MELD score, blood loss, dialysis before transplant, hepatic artery thrombosis, and nonanastomotic strictures were associated with graft loss, and laboratory MELD score, dialysis before transplant, and blood loss were associated with patient survival. In the multivariable models, dialysis at transplant (hazard ratio [HR], 8.04 [95% CI, 2.52-25.61]; *P* < .001), hepatic artery thrombosis (HR, 13.49 [95% CI, 4.73-38.45]; *P* < .001), and nonanastomotic strictures (HR, 9.61 [95% CI, 3.80-24.30]; *P* < .001) remained independently associated with graft loss, whereas dialysis at transplant was the only variable independently associated with patient survival (HR, 9.47 [95% CI, 2.38-37.64]; *P* < .001). Use of DHOPE-PRO was not associated with either graft (HR, 1.28 [95% CI, 0.59-2.74]; *P* = .53) or patient (HR, 2.05 [95% CI, 0.75-5.59]; *P* = .16) survival (eFigure 5 in Supplement 1).

## Discussion

This prospective cohort study assessed program-level implementation and clinical outcomes of liver transplant following routine implementation of DHOPE-PRO. By extending preservation time, DHOPE-PRO was associated with completion of 89.1% of liver transplants during daytime hours and with outcomes similar to those following short-duration DHOPE. To our knowledge, this was the first prospective, practice-based evaluation of DHOPE-PRO focused on procedural timing and intermediate-term outcomes.

Our findings indicated that DHOPE-PRO may be applied to both DBD and DCD grafts for durations up to 20.5 hours and 23.8 hours, respectively, with total preservation times of up to 26.3 and 31.4 hours, respectively, without increases in transplant-related complications. This study builds on prior evidence from the DHOPE-PRO trial, which focused on DBD grafts and compared short vs prolonged DHOPE.^21^ We extended those insights to include both standard and ECD-DCD grafts and provide new comparative data between DHOPE-PRO and no perfusion in DBD transplant. Subgroup analyses revealed no significant differences in intraoperative variables, intensive care unit or hospital length of stays, vascular or biliary complications, graft survival, or patient survival.

While there was a higher proportion of standard DCD graft recipients in the DHOPE-PRO group requiring temporary new-onset RRT, the overall incidence of AKI severity or AKI duration was not significantly different between groups. However, the possibility of a type II error cannot be excluded. Furthermore, there was no correlation between peak transaminase levels, duration of DHOPE, and total preservation time across subgroups (eFigure 4 in Supplement 1). Similarly, routinely prolonging DHOPE for up to 24 hours was not associated with posttransplant complications compared with short-duration DHOPE for DCD or no machine perfusion for DBD liver transplants.

With the implementation of DHOPE-PRO, liver transplant may no longer need to be performed immediately after organ retrieval, allowing flexibility in addressing logistical, surgical, and technical challenges (Figure 1). This approach enabled us to schedule liver transplants during daytime hours, thereby improving surgical workflow and reducing the need for overnight operations. Avoiding nighttime surgical procedures may support staff well-being by reducing fatigue and improving work-life balance.

DHOPE-PRO offered logistical advantages including enabling reallocation of a donor liver when recipient changes were required, without compromising graft quality, and allowing for controlled timing of combined organ procedures (eg, heart-liver or lung-liver transplant) (Figure 3C). Additionally, DHOPE-PRO facilitated ex situ liver splitting in the perfusion machine (Figure 3B), avoiding additional ischemic injury, and was associated with reduced risk of ischemic injury in technically challenging cases with anticipated prolonged hepatectomy times, such as complex retransplant procedures.^30^ Collectively, these benefits contribute to a more efficient, sustainable, and resilient transplant program, without compromising clinical outcomes.

A recent retrospective cohort study from Italy included 177 DHOPE perfusions lasting more than 4 hours across 12 centers and compared outcomes using propensity score matching with 177 DHOPE perfusions of up to 4 hours.^31^ Median DHOPE perfusion and total preservation times in that study were 5 and 10 hours, respectively. These durations were substantially shorter than those in the present study, in which the median DHOPE perfusion and total preservation times were nearly double (10.2 and 18.6 hours, respectively). The Italian study reported a significantly lower incidence of AKI after transplant in the group that received a liver after prolonged DHOPE compared with short DHOPE (median DHOPE duration of 2 hours). This finding could not be replicated in the current study and warrants further investigation.

NMP is also increasingly used for logistical purposes. NMP is often initiated at the donor hospital and maintained during transport to the transplantation center via dedicated device-to-donor service. This method of continuous NMP has facilitated daytime transplant in up to 89% of cases, albeit at significantly higher costs (more than $47 000 US for index hospitalization, largely driven by increased organ acquisition costs of $85 000 US).^32^ Other centers use a back-to-base approach, whereby donor livers undergo end-ischemic NMP at the receiving transplant center prior to transplant. This method has also successfully extended preservation times and reduced the frequency of nighttime surgical procedures without negatively impacting outcomes.^33^

Although NMP can safely extend preservation time and improve early posttransplant outcomes, its clinical implementation incurs significantly higher costs.^32,33,34^ In contrast, the UMCG Comprehensive Transplant Center has adopted DHOPE-PRO as a more pragmatic strategy to achieve similar logistical gains. By preserving the graft in a metabolically quiescent state at low temperature and low perfusion pressures, DHOPE ensures a greater safety margin during perfusion and minimizes the risk of device-related complications.^35,36^ Unlike NMP, DHOPE has been associated with reduced ischemia-reperfusion injury and lower rates of complications after transplant, such as ischemic cholangiopathy, compared with static cold storage.^4,5,6,7,8,9,10^ Our findings indicated that even with total preservation times extending up to 31.4 hours, prolonged DHOPE maintained clinical safety and facilitated daytime transplant procedures. These advantages underscore our choice to prioritize DHOPE-PRO over NMP for logistical purposes, not due to limitations in efficacy, but due to its simplicity, reliability, and cost-effectiveness in routine clinical practice. In this context, we support the selective use of NMP following DHOPE(-PRO) and COR, where NMP serves as a valuable tool for viability assessment of high-risk, ECD grafts.^18,37^

### Strengths and Limitations

This study has strengths. It was prospectively designed and included all consecutive liver transplants performed over a defined period, with structured subgroup analyses spanning multiple graft types and preservation strategies. The completeness of inclusion, standardized clinical protocols, and systematic data collection enhanced the internal validity of our findings.

Several limitations should be acknowledged. While DHOPE-PRO was applied for up to 24 hours, with total preservation times extending to 31.4 hours, the optimal and maximal safe durations remain unknown and were not systematically evaluated. However, no correlation was observed between DHOPE duration and postoperative transaminase levels. The current follow-up period did not allow for evaluation of biliary complications beyond 12 months, immunological rejection, or patient-reported outcomes. Furthermore, this was a single-center study; although the structured implementation and consistent outcomes suggest broader implementation, external validation is required. Applicability may also vary across regions due to differences in donor logistics and the availability of specialized perfusion expertise. Future multicenter studies with longer follow-up are necessary to evaluate the long-term safety, efficacy, and generalizability of this approach.

## Conclusions

In this prospective cohort study, routine implementation of DHOPE-PRO was associated with an increased proportion of daytime liver transplants and with improved surgical logistics as well as with outcomes similar to those after short-duration DHOPE. These findings underscore the potential of DHOPE-PRO as an organ preservation strategy and provide supporting evidence for its broader adoption in clinical practice.

## Notes

Supplement 1. **eFigure 1.** Flow diagram showing the number and type of livers included in the study referring to the program level comparison **eFigure 2.** Distribution of machine perfusion times before (2021-2022) and after (2023-2024) implementation of routine DHOPE-PRO **eFigure 3.** Case of pediatric DHOPE-PRO **eFigure 4.** Post-transplant AST and ALT measurements **eFigure 5.** Multivariate regression analysis of recipient factors influencing development of new onset AKI and new onset RRT, and multivariate Cox regression analysis of recipient factors influencing graft and patient survival **eTable.** Donor and perfusion characteristics

Supplement 2.
The DHOPE-PRO Investigators

Supplement 3.
Data Sharing Statement
