# Supplementary material for: Prolonged Dual Hypothermic Oxygenated Machine Perfusion for Daytime Liver Transplant
Source: JAMA Netw Open. 2026 Apr 2;9(4):e265039. doi: 10.1001/jamanetworkopen.2026.5039 (PMC13047461; doi:10.1001/jamanetworkopen.2026.5039)
Supplement: Supplement 1. — eFigure 1. Flow diagram showing the number and type of livers included in the study referring to the program level comparison eFigure 2. Distribution of machine perfusion times before (2021-2022) and after (2023-2024) implementation of routine DHOPE-PRO eFigure 3. Case of pediatric DHOPE-PRO eFigure 4. Post-transplant AST and ALT measurements eFigure 5. Multivariate regression analysis of recipient factors influencing development of new onset AKI and new onset RRT, and multivariate Cox regression analysis of recipient factors influencing graft and patient survival eTable. Donor and perfusion characteristics [file jamanetwopen-e265039-s001.pdf]

Supplementary Online Content

Bodewes SB, Woltjes LC, Thorne AM, et al; for the DHOPE-PRO Investigators. Prolonged dual hypothermic oxygenated machine perfusion for daytime liver transplant. *JAMA Netw Open*. 2026;9(4):e265039. doi:10.1001/jamanetworkopen.2026.5039

eFigure 1. Flow diagram showing the number and type of livers included in the study referring to the program level comparison

eFigure 2. Distribution of machine perfusion times before (2021-2022) and after (2023-2024) implementation of routine DHOPE-PRO

eFigure 3. Case of pediatric DHOPE-PRO

eFigure 4. Post-transplant AST and ALT measurements

eFigure 5. Multivariate regression analysis of recipient factors influencing development of new onset AKI and new onset RRT, and multivariate Cox regression analysis of recipient factors influencing graft and patient survival

eTable. Donor and perfusion characteristics

This supplementary material has been provided by the authors to give readers additional information about their work.

© 2026 Bodewes SB et al. *JAMA Network Open*.

eFigure 1. Flow diagram showing the number and type of livers included in the study referring to the program level comparison

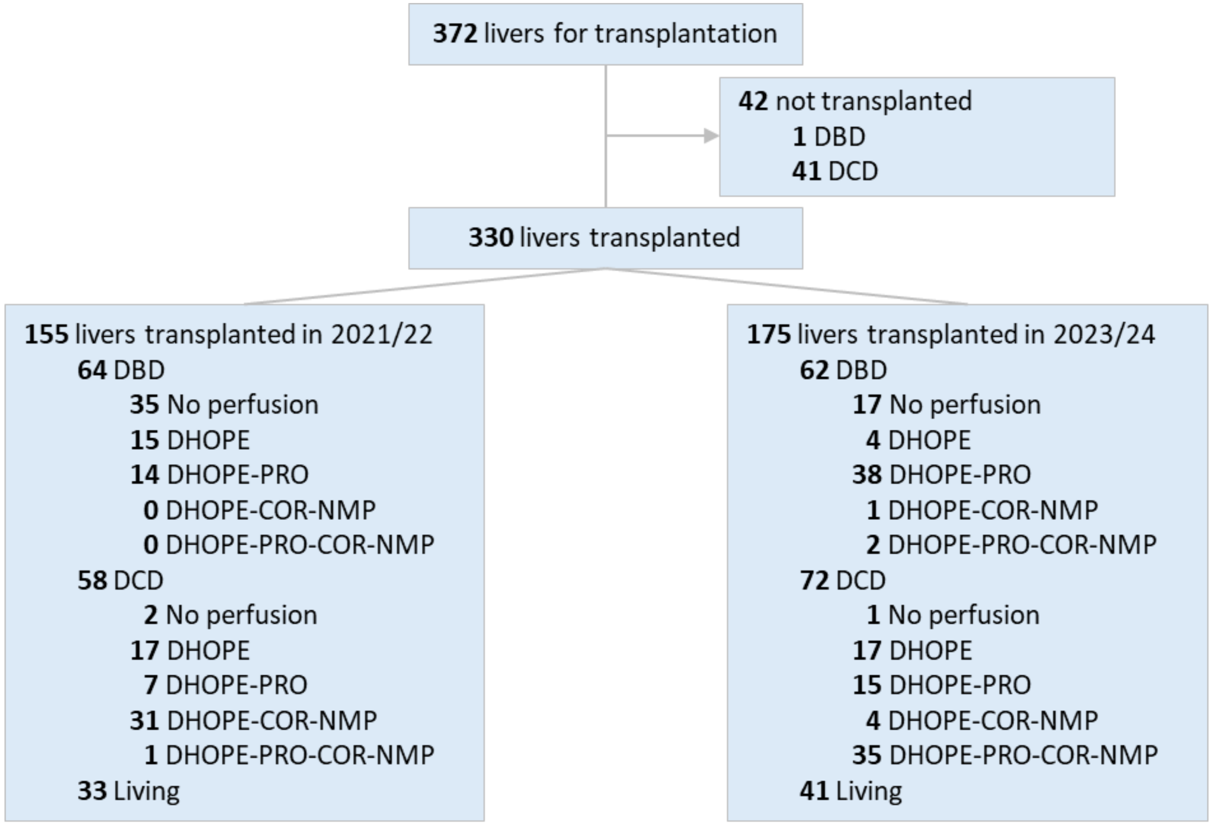

© 2026 Bodewes SB et al. *JAMA Network Open*.

eFigure 2. Distribution of machine perfusion times before (2021-2022) and after (2023-2024) implementation of routine DHOPE-PRO. (A) Range plot depicting times (HH:MM) of start of perfusion for DHOPE only and DHOPE-COR-NMP protocols. (B) Bar chart showing the distribution of daytime (08:00-20:00) and nighttime (20:00-08:00) end of perfusion. COR; controlled, oxygenated rewarming, DHOPE; dual hypothermic oxygenated machine perfusion, PRO; prolonged perfusion.

© 2026 Bodewes SB et al. *JAMA Network Open*.

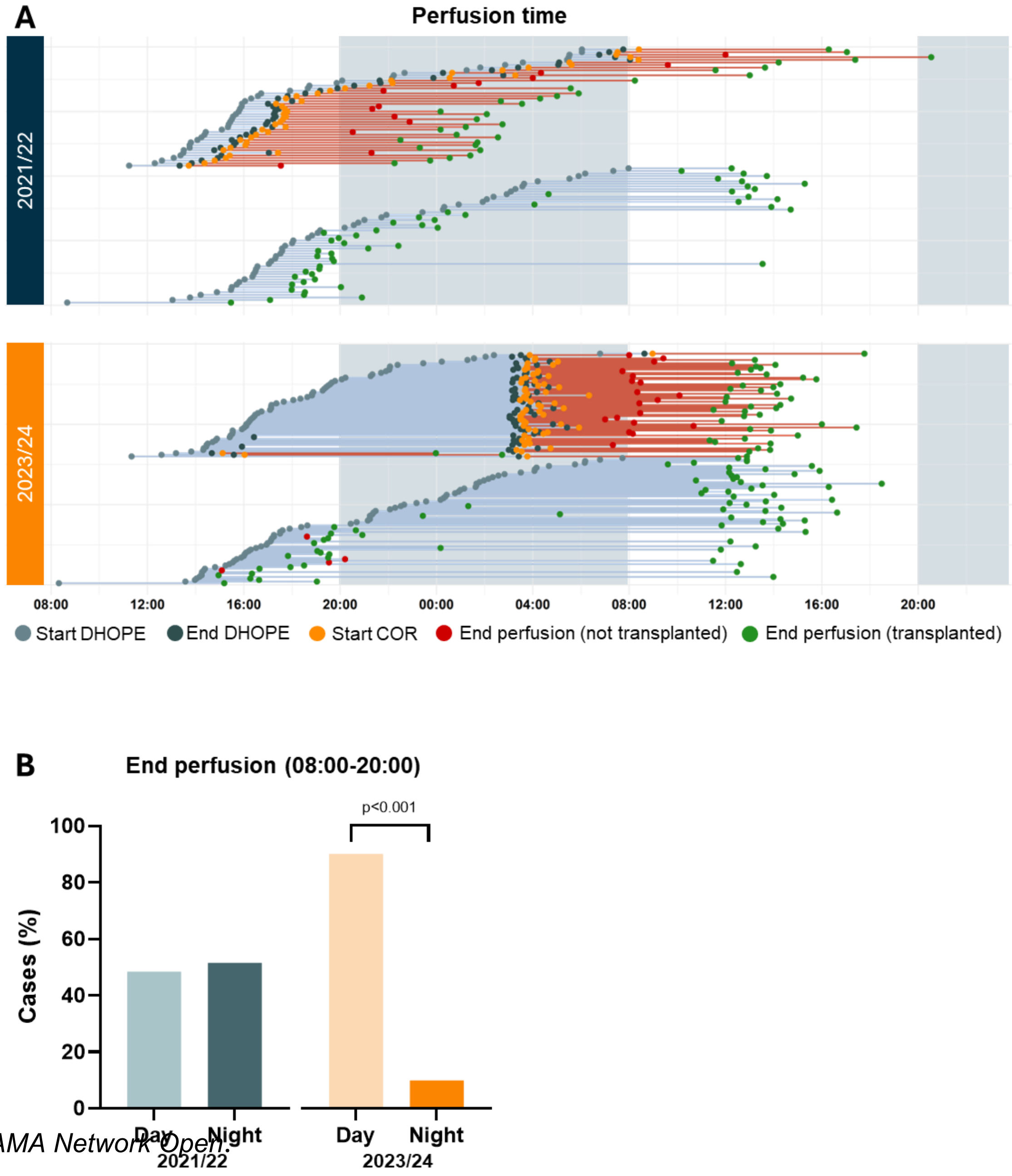

© 2026 Bodewes SB et al. *JAMA Network Open*.

eFigure 3. Cases of pediatric DHOPE-PRO. (A) Photo of a liver graft (760 g) from a 4-year-old DBD donor which underwent DHOPE-PRO to facilitate complex pediatric retransplantation. (B) Liver graft (291 g) from a 3-month-old DBD donor underwent single HOPE-PRO due to the technical complexity of size-matched transplantation.

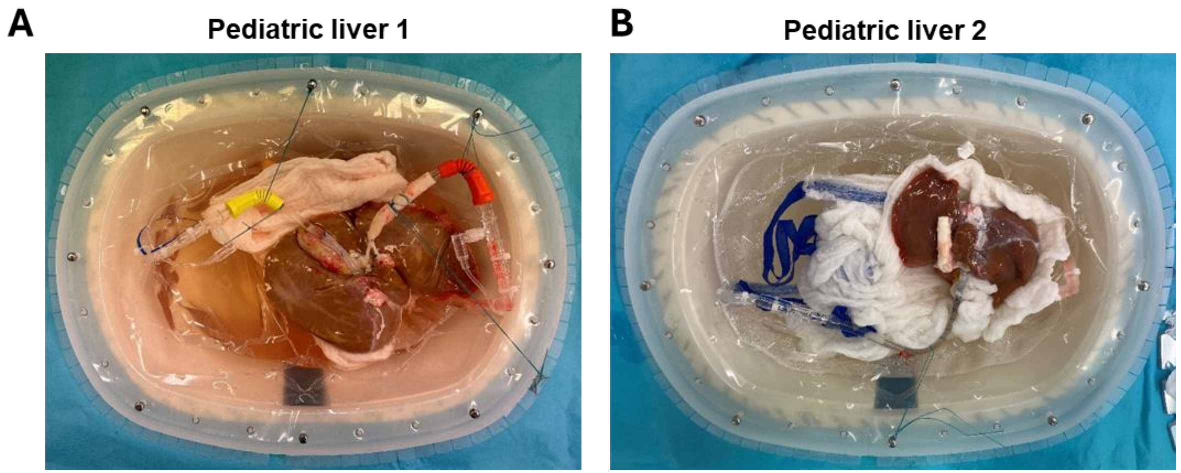

© 2026 Bodewes SB et al. *JAMA Network Open*.

eFigure 4. Post-transplant AST and ALT measurements

AST measurements during post-transplant in (A) Standard DBD: No perfusion vs DHOPE-PRO, (B) Standard DCD: DHOPE vs DHOPE-PRO and (C) DCD that underwent viability assessment: DHOPE-COR-NMP vs DHOPE-PRO-COR-NMP. Correlation plots of post-transplant peak AST and ALT compared to (cold) preservation time. (D) Standard DBD: No perfusion, (E) Standard DCD: DHOPE, (F) DCD that underwent viability assessment: DHOPE-COR-NMP, (G) Standard DBD: DHOPE-PRO, (H) Standard DCD: DHOPE-PRO and (I) DCD that underwent viability assessment: DHOPE-PRO-COR-NMP.

© 2026 Bodewes SB et al. *JAMA Network Open*.

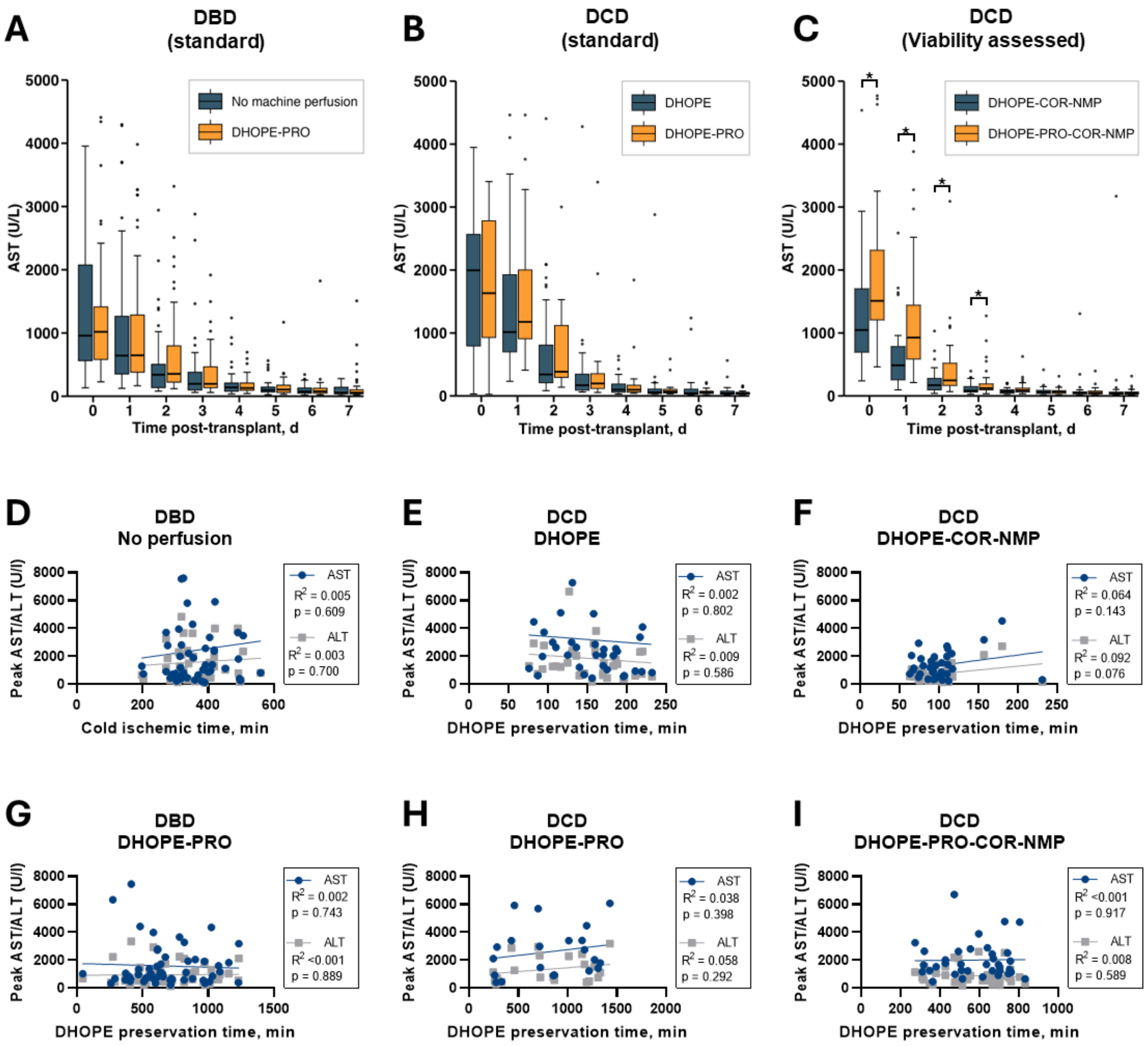

© 2026 Bodewes SB et al. *JAMA Network Open*.

eFigure 5. Multivariate regression analysis of recipient factors influencing development of (A) new onset AKI and (B) new onset RRT. Multivariate Cox regression analysis of recipient factors influencing (C) graft and (D) patient survival. AKI; acute kidney injury, RRT; renal replacement therapy.

© 2026 Bodewes SB et al. *JAMA Network Open*.

A

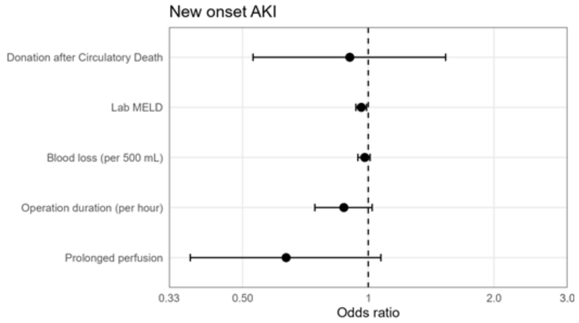

Effect	Odds ratio (CI)	P value
Donation after Circulatory Death	0.90 (0.53–1.53)	0.71
Lab MELD	0.96 (0.93–0.99)	0.01
Blood loss (per 500 mL)	0.98 (0.94–1.01)	0.25
Operation duration (per hour)	0.87 (0.74–1.02)	0.09
Prolonged perfusion	0.64 (0.37–1.07)	0.09

B

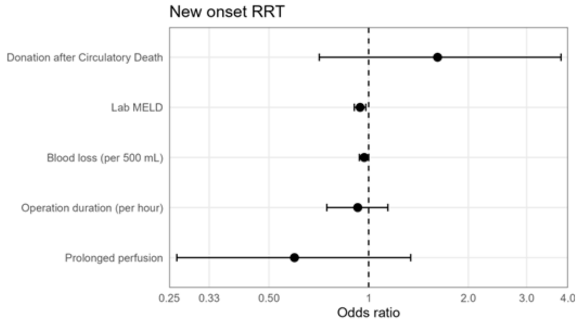

Effect	Odds ratio (CI)	P value
Donation after Circulatory Death	1.62 (0.71–3.81)	0.26
Lab MELD	0.94 (0.90–0.98)	0.00
Blood loss (per 500 mL)	0.97 (0.94–1.00)	0.05
Operation duration (per hour)	0.93 (0.75–1.14)	0.48
Prolonged perfusion	0.60 (0.26–1.34)	0.21

C

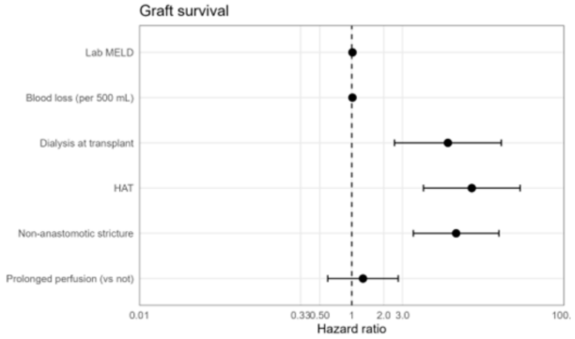

Effect	Hazard ratio (CI)	P value
Lab MELD	1.02 (0.97–1.06)	0.50
Blood loss (per 500 mL)	1.01 (1.00–1.03)	0.12
Dialysis at transplant	8.04 (2.52–25.61)	<0.001
HAT	13.49 (4.73–38.45)	<0.001
Prolonged perfusion (vs not)	1.28 (0.59–2.74)	0.53
Non-anastomotic stricture	9.61 (3.80–24.30)	<0.001

D

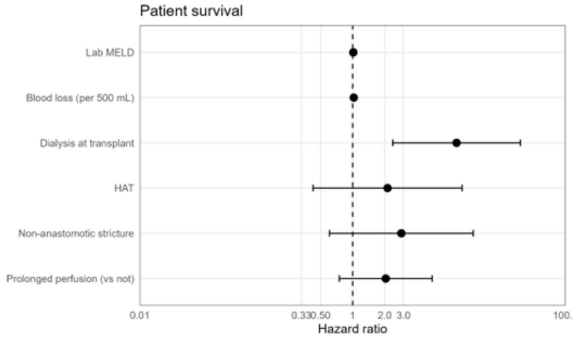

Effect	Hazard ratio (CI)	P value
Lab MELD	1.02 (0.96–1.07)	0.61
Blood loss (per 500 mL)	1.03 (1.00–1.05)	0.05
Dialysis at transplant	9.47 (2.38–37.64)	<0.001
HAT	2.13 (0.42–10.69)	0.36
Prolonged perfusion (vs not)	2.05 (0.75–5.59)	0.16
Non-anastomotic stricture	2.87 (0.61–13.58)	0.18

© 2026 Bodewes SB et al. *JAMA Network Open*.

eTable. Donor and perfusion characteristics

	2021/22 (n=155)	2023/24 (n=174)	p-value
Donor characteristics			
Age (years)	48 (34-61)	48 (33-63)	.78
Body mass index (kg/m^2)	25 (22-27)	25 (22-28)	.30
Gender (%)			
Male	86 (56)	95 (55)	.96
Female	69 (45)	79 (45)	
Donor type (%)			
DCD	58 (37)	72 (41)	.51
DBD	64 (41)	61 (35)	
Living	33 (21)	41 (24)	
Cause of death (%)			
Trauma	20 (13)	23 (13)	
CVA	37 (24)	41 (24)	.79
Anoxia	31 (20)	26 (15)	
Other	34 (22)	43 (25)	
Time from withdrawal of life support to circulatory arrest (min)	14 (10-16)	14 (10-18)	.24
Time from circulatory arrest to cold perfusion (min)	16 (15-19)	16 (15-19)	.17
Functional donor warm ischemia time (min)	28 (23-33)	27 (24-33)	.59
Last AST (U/L)	41 (25-97)	49 (27-79)	.31
Last ALT (U/L)	37 (20-77)	33 (21-58)	.31
Last GGT (U/L)	35 (19-82)	38 (21-83)	.66
Hepatectomy time (min)	33 (29-42)	33 (28-41)	.83
Static cold ischemia time (hours)	4.2 (3.6-4.8)	4.1 (3.7-4.6)	.91
DRI	2.24 (1.71-2.66)	2.25 (1.77-2.72)	.65
Perfusion characteristics§			
Type of perfusion (%)			
No perfusion	70 (45)	59 (34)	**<0.001**
Post-mortal	37 (23)	18 (10)	

© 2026 Bodewes SB et al. *JAMA Network Open*.

Living	33 (21)	41 (23)	
Standard machine perfusion	63 (41)	26 (15)	
(D)HOPE	32 (21)	21 (12)	
(D)HOPE-COR-NMP	31 (20)	5 (2.9)	
Prolonged machine perfusion	22 (14)	90 (51)	
(D)HOPE-PRO	21 (14)	53 (30)	
(D)HOPE-PRO-COR-NMP	1 (0.6)	37 (21)	
Duration (D)HOPE (hours)	2.1 (1.6-4.1)	10.2 (5.1-13.1)	**<0.001**
Duration COR-NMP (hours)	9.4 (8.3-10.0)	9.2 (8.6-9.9)	.27
Duration machine perfusion (hours)	7.8 (2.6-10.7)	14.3 (7.8-19.3)	**<0.001**
Total preservation time (hours)	9.3 (7.3-15.3)	18.6 (9.1-24.6)	**<0.001**

Continuous data are presented as median (IQR), categorical data as a number (percentage). [§]2023/24 includes a split liver, of which both lobes were perfused and transplanted, resulting in one more in perfusion count than donor count. ALP; alkaline phosphatase, ALT; alanine aminotransferase, AST; aspartate aminotransferase, CVA; cerebral vascular accident, DBD; donation after brain death, DCD; donation after circulatory death, DRI; Donor risk index

© 2026 Bodewes SB et al. *JAMA Network Open*.
