# Supplementary material for: Prolonged Dual Hypothermic Oxygenated Machine Perfusion for Daytime Liver Transplant
Source: JAMA Netw Open. 2026 Apr 2;9(4):e265039. doi: 10.1001/jamanetworkopen.2026.5039 (PMC13047461; doi:10.1001/jamanetworkopen.2026.5039)
Supplement: Supplement 2. — The DHOPE-PRO Investigators [file jamanetwopen-e265039-s002.pdf]

Supplemental Online Content: Nonauthor Collaborators
*First name, last name, and suffix (if applicable) are required and will appear in PubMed.

*Group Name(s): DHOPE-PRO Investigators							
***First Name and Middle Initial(s)**	***Last Name**	***Suffix (eg, Jr, III)**	Academic Degrees	Institution	Location (city, state/province, country)	Role or Contribution, eg, chair, principal investigator	Group (if more than 1 Group listed in the byline) and/or Subgroup (eg, Steering Committee)
Marieke T.	de Boer			University Medical Center Groningen	Groningen, the Netherlands	Transplant surgeon	
Carlijn I.	Buis			University Medical Center Groningen	Groningen, the Netherlands	Transplant surgeon	
Suomi M.G.	Fouraschen			University Medical Center Groningen	Groningen, the Netherlands	Transplant surgeon	
Hermien	Hartog			University Medical Center Groningen	Groningen, the Netherlands	Transplant surgeon	
Frederik J.H.	Hoogwater			University Medical Center Groningen	Groningen, the Netherlands	Transplant surgeon	
Vincent E.	de Meijer			University Medical Center Groningen	Groningen, the Netherlands	Transplant surgeon	
Joost M.	Klaase			University Medical Center Groningen	Groningen, the Netherlands	Transplant surgeon	
Ruben H.J.	de Kleine			University Medical Center Groningen	Groningen, the Netherlands	Transplant surgeon	
Mark	Meerdink			University Medical Center Groningen	Groningen, the Netherlands	Transplant surgeon	
Maarten W.	Nijkamp			University Medical Center Groningen	Groningen, the Netherlands	Transplant surgeon	
Hans	Blokzijl			University Medical Center Groningen	Groningen, the Netherlands	Transplant hepatologist	
Frans J.C.	Cuperus			University Medical Center Groningen	Groningen, the Netherlands	Transplant hepatologist	
Frans	van der Heide			University Medical Center Groningen	Groningen, the Netherlands	Transplant hepatologist	
Daphne M.	Hotho			University Medical Center Groningen	Groningen, the Netherlands	Transplant hepatologist	

Supplemental Online Content: Nonauthor Collaborators
*First name, last name, and suffix (if applicable) are required and will appear in PubMed.

***First Name and Middle Initial(s)**	***Last Name**	***Suffix (eg, Jr, III)**	Academic Degrees	Institution	Location (city, state/province, country)	Role or Contribution, eg, chair, principal investigator	Group (if more than 1 Group listed in the byline) and/or Subgroup (eg, Steering Committee)
Frederike G.I.	van Vilsteren			University Medical Center Groningen	Groningen, the Netherlands	Transplant hepatologist	
A. Boudewijn	de Vries			University Medical Center Groningen	Groningen, the Netherlands	Transplant hepatologist	
Ilhama F.	Abbasova			University Medical Center Groningen	Groningen, the Netherlands	Transplant anesthesiologist	
Jop P.	van den Berg			University Medical Center Groningen	Groningen, the Netherlands	Transplant anesthesiologist	
Jan-Willem H.L.	Boldingh			University Medical Center Groningen	Groningen, the Netherlands	Transplant anesthesiologist	
Meine H.	Fernhout			University Medical Center Groningen	Groningen, the Netherlands	Transplant anesthesiologist	
Manon W.J.	van der Heijden			University Medical Center Groningen	Groningen, the Netherlands	Transplant anesthesiologist	
Ernesto R.R.	Muskiet			University Medical Center Groningen	Groningen, the Netherlands	Transplant anesthesiologist	
Koen M.E.M.	Reyntjens			University Medical Center Groningen	Groningen, the Netherlands	Transplant anesthesiologist	
Jaap J.	Vos			University Medical Center Groningen	Groningen, the Netherlands	Transplant anesthesiologist	
Miriam	Zeillemaker			University Medical Center Groningen	Groningen, the Netherlands	Transplant anesthesiologist	
Isabel M.A.	Brüggenwirth			University Medical Center Groningen	Groningen, the Netherlands	Co-investigator, perfusionist	
Veerle A.	Lantinga			University Medical Center Groningen	Groningen, the Netherlands	Co-investigator, perfusionist	
Bianca	Lascaris			University Medical Center Groningen	Groningen, the Netherlands	Organ perfusionist	
Adam M.	Thorne			University Medical Center Groningen	Groningen, the Netherlands	Co-investigator, perfusionist	

Supplemental Online Content: Nonauthor Collaborators
*First name, last name, and suffix (if applicable) are required and will appear in PubMed.

***First Name and Middle Initial(s)**	***Last Name**	***Suffix (eg, Jr, III)**	Academic Degrees	Institution	Location (city, state/province, country)	Role or Contribution, eg, chair, principal investigator	Group (if more than 1 Group listed in the byline) and/or Subgroup (eg, Steering Committee)
Silke B.	Bodewes			University Medical Center Groningen	Groningen, the Netherlands	Co-investigator, perfusionist	
Ton	Lisman			University Medical Center Groningen	Groningen, the Netherlands	Co-investigator	
Wouke N.	Kuiper			University Medical Center Groningen	Groningen, the Netherlands	Organ perfusionist	
Vivianne E.	Veenma			University Medical Center Groningen	Groningen, the Netherlands	Organ perfusionist	
Minou	de Bree			University Medical Center Groningen	Groningen, the Netherlands	Organ perfusionist	
Kirsten F.	Ma			University Medical Center Groningen	Groningen, the Netherlands	Organ perfusionist	
Roman	Kalashnik			University Medical Center Groningen	Groningen, the Netherlands	Organ perfusionist	
Giel M.	Van Vliet			University Medical Center Groningen	Groningen, the Netherlands	Organ perfusionist	
Kees	Wieriks			University Medical Center Groningen	Groningen, the Netherlands	Organ perfusionist	
Carin	Biel			University Medical Center Groningen	Groningen, the Netherlands	Organ perfusionist	
C. Willemijn	Binnekamp			University Medical Center Groningen	Groningen, the Netherlands	Organ perfusionist	
