# Supplementary material for: Prolonged Dual Hypothermic Oxygenated Machine Perfusion for Daytime Liver Transplant
Source: JAMA Netw Open. 2026 Apr 2;9(4):e265039. doi: 10.1001/jamanetworkopen.2026.5039 (PMC13047461; doi:10.1001/jamanetworkopen.2026.5039)
Supplement: Supplement 3. — Data Sharing Statement [file jamanetwopen-e265039-s003.pdf]

Data Sharing Statement

Bodewes. Prolonged Dual Hypothermic Oxygenated Machine Perfusion for Daytime Liver Transplant. *JAMA Netw Open*. Published April 02, 2026.
doi:10.1001/jamanetworkopen.2026.5039

Data
Data available: No

Additional Information
Explanation for why data not available: Collated, de-identified patient data will be made available upon reasonable request to the corresponding author. Requests will require institutional review board approval and a signed data transfer agreement.
